# Nerve Restoration

**Published:** 1890-10

**Authors:** 


### Nerve Restoration.

Dr. Gluck reported a case before a medical society lately of a man who was stabbed on the outside of the forearm with a table knife in August, 1887. The wound was treated in the usual way, but in September complete paralysis of the parts supplied by the radial nerve was found to have taken place. An attempt was then made to reunite the ends of the divided nerve. After careful search for the peripheral and centripetal ends, it was found that they were five ctm. apart, and that they could not be brought together by any possible means. The ends were then freshened and loosened from their surroundings, and indirectly united by means of catgut loops. Healing took place in ten days. Electrical treatment was then carried out under the direction of Prof. Bernhardt. In the course of a year complete restoration of function had taken place, which the speaker observed could only have been effected by actual growth of nerve elements along the tract of the catgut thread.—*Med. Press and Circular.*

